# Role of Smad3 and p38 Signalling in Cigarette Smoke-induced CFTR and BK dysfunction in Primary Human Bronchial Airway Epithelial Cells

**DOI:** 10.1038/s41598-017-11038-x

**Published:** 2017-09-05

**Authors:** Juliette Sailland, Astrid Grosche, Nathalie Baumlin, John S. Dennis, Andreas Schmid, Stefanie Krick, Matthias Salathe

**Affiliations:** 1 Division of Pulmonary, Allergy, Critical Care and Sleep Medicine, University of Miami Miller School of Medicine, Miami, FL 33136 USA; 2 Present Address: Division of Pulmonary, Allergy & Critical Care Medicine, University of Alabama at Birmingham, Birmingham, AL 35294 USA; 3 Present Address: Department of Radiation Oncology, University of Florida, Gainesville, FL 32610 USA

## Abstract

Mucociliary clearance (MCC) is a major airway host defence system that is impaired in patients with smoking-associated chronic bronchitis. This dysfunction is partially related to a decrease of airway surface liquid (ASL) volume that is in part regulated by apically expressed cystic fibrosis transmembrane conductance regulator (CFTR) and large-conductance, Ca^2+^-activated, and voltage dependent K^+^ (BK) channels. Here, data from human bronchial epithelial cells (HBEC) confirm that cigarette smoke not only downregulates CFTR activity but also inhibits BK channel function, thereby causing ASL depletion. Inhibition of signalling pathways involved in cigarette smoke-induced channel dysfunction reveals that CFTR activity is downregulated via Smad3 signalling whereas BK activity is decreased via the p38 cascade. In addition, pre-treatment with pirfenidone, a drug presently used to inhibit TGF-β signalling in idiopathic pulmonary fibrosis, ameliorated BK dysfunction and ASL volume loss. Taken together, our results highlight the importance of not only CFTR but also BK channel function in maintaining ASL homeostasis and emphasize the possibility that pirfenidone could be employed as a novel therapeutic regimen to help improve MCC in smoking-related chronic bronchitis.

## Introduction

The airway epithelium represents the lung’s first line of defence^1^ and constitutes an essential protection to inhaled insults. Mucociliary clearance (MCC) is the primary mechanism for removing inhaled noxious stimuli. Effective MCC relies upon several components, including ciliary beating and airway surface liquid (ASL), responsible for adequate mucus hydration. Various ion fluxes across the apical membrane control transepithelial water flow and thus regulate ASL volume^2–5^. In this context, a balance of sodium (Na^+^) absorption and chloride (Cl^−^) secretion has been implicated to be most important^6^. Chloride movement across polarized epithelia is in part controlled by the cystic fibrosis transmembrane conductance regulator (CFTR) channel that is critical to regulate airway fluid homeostasis and maintain functional ciliary beating^7, 8^. However, apical potassium (K^+^) secretion is increasingly recognized to play a role in providing an electrochemical driving force for apical Cl^−^ exit through CFTR and Calcium-activated chloride channels (CaCC)^9^. In fact, when BK channel function is diminished in normal primary human bronchial epithelial cells (HBECs), either by inhibitors or by BK α subunit knockdown, the epithelial surface dries out, making apical BK function instrumental for the maintenance and regulation of ASL volume and MCC^10–13^.

Smoking impairs MCC^14, 15^ and is a major risk factor for the pathogenesis of chronic bronchitis and chronic obstructive pulmonary disease (COPD)^16, 17^, both associated with increased morbidity and mortality^18^. Cigarette smoke has been shown to significantly reduce CFTR-mediated Cl^−^ secretion *in vitro* and *in vivo* ^8, 19–22^. Furthermore, p38 mitogen-activated protein kinase (MAPK) signalling is activated by tobacco smoke and its activation is associated with COPD^23^. While p38 MAPK regulates the expression of the epithelial sodium channel ENaC^24^, its role in regulating CFTR and BK channel activities is ambiguous. In the kidney, inhibition of p38 MAPK causes activation of BK in principal and intercalated cells^25^. On the other hand, cigarette smoke extract exposure of a human airway epithelial cell line has been reported to regulate the loss of plasma membrane CFTR via MEK/ERK/MAPK but not via p38^26^.

Aside from p38, transforming growth factor beta 1 (TGF-β1) is upregulated in small airway epithelia of COPD patients. There, TGF-β1 levels correlate with the severity of obstruction^27–29^. In cystic fibrosis (CF), TGF-β1 has been described to cause mucociliary dysfunction by reducing ASL volume via decreased apical BK channel activity^11^ through a down-regulation of Leucine Rich Repeat Containing protein 26 (LRRC26), the γ subunit necessary for BK function in non-excitable tissues^30^. By activating cell surface receptors, TGF-β induces Smad2/Smad3 phosphorylation, which triggers the translocation of the complex to the nucleus where it affects chromatin remodelling and the transcription of target responsive genes^31^. Moreover, TGF-β activates other pathways independently of Smad signalling, such as Jun N-terminal kinase (JNK), p38 MAPK, ERK or MEKK^32^.

In the present study, we tested the hypothesis that activation of canonical Smad3 and p38 MAPK signalling by cigarette smoke differentially affects CFTR and BK channel functions and addressed whether either channel can uphold ASL volume homeostasis in HBECs.

## Results

### Cigarette smoke causes CFTR and BK channel dysfunction and impairs ASL volume in HBECs

**Figure 1:**
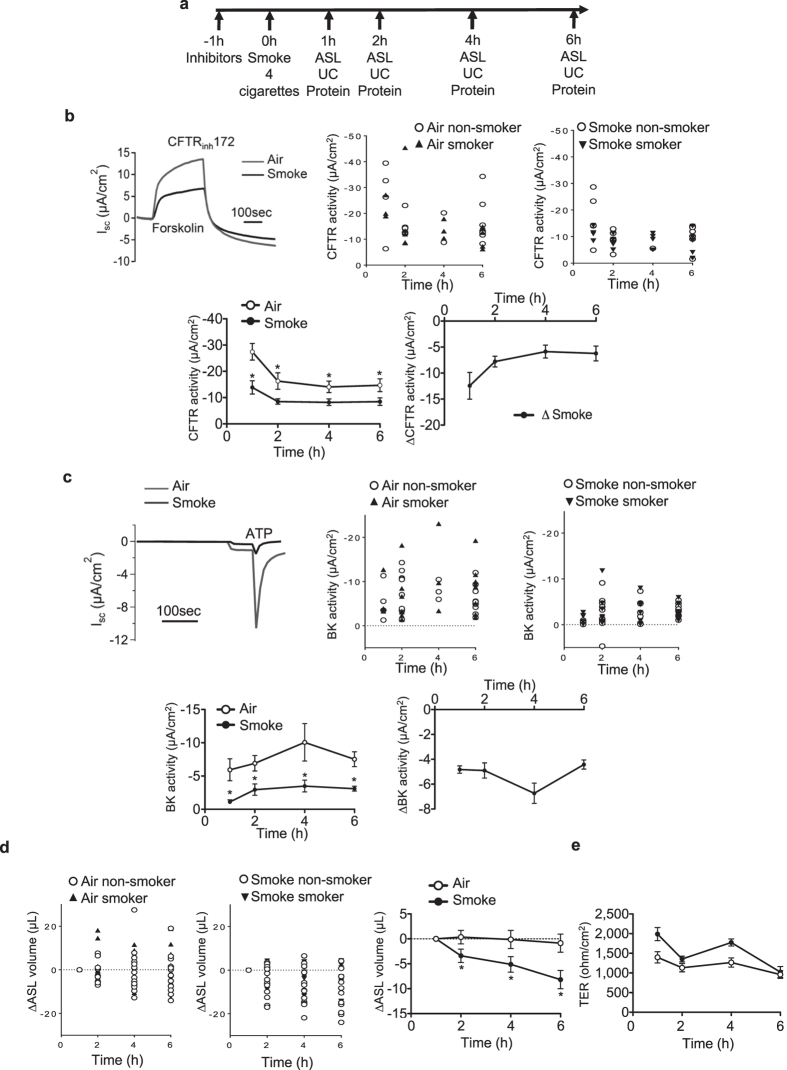
Effect of tobacco smoke on MCC parameters *in vitro*. **(a)** Graph showing the experimental design. Abbreviations are: ASL (airway surface liquid), UC (Ussing chamber). **(b)** Upper left: representative traces of 10 µM CFTR_inh_172-induced short circuit current (Isc) changes after 10 µM forskolin stimulation of HBECs exposed to 10 µM amiloride. Tobacco smoke exposure of fully differentiated HBECs via the VC-10 smoking robot using 24 puffs of a total of 4 cigarettes (Kentucky 3R4F) reduced CFTR conductance 1 h after cigarette smoke exposure. Middle and upper right: Representation of CFTR channel activities of cells from smokers and non-smokers exposed to the air (middle) and smoke (right). Lower left: time course of CFTR activity after smoke or air exposure. Lower right: Decreased CFTR activity is represented by the different channel activities of smoked cells and cells exposed to air (ΔCFTR activity): ΔSmoke = CFTR activity of smoke-exposed cells – control cells exposed to air. **(c)** Upper left: Representative traces of ATP-induced (10 µM) short circuit current (Isc) changes in basolaterally permeabilized HBECs cells exposed to a basolateral-to apical K^+^ gradient in the presence of 10 µM amiloride. Middle and upper right: Representation of BK channel activities of cells from smokers and non-smokers exposed to air (middle) and smoke (right). Lower left: Cigarette smoke exposure decreased BK activity within 1 h of exposure (ATP elicited Isc after basolateral permeabilization and a basolateral to apical K^+^ gradient). Lower right: BK activity decrease is represented by the channel activity difference of smoke-exposed cells compared to air-exposed cells (ΔBK activity): ΔSmoke = BK activity of smoke-exposed cells – cells exposed to the air. **(d)** Smoke reduces ASL volume 2 h after exposure (meniscus scanning method) but **(e)** no change in transepithelial resistance (TER) is seen. All n ≥ 4 from ≥ 3 different lung donors. * indicates p < 0.05 compared to air exposure at the same time point.

To examine the effects of cigarette smoke on BK and CFTR activity and ASL volume, primary HBECs from both non-smokers and smokers were cultured at the air-liquid interface to full differentiation^8, 33–36^. Cells were pre-treated with different pathway inhibitors 1 h prior exposure to smoke (4 cigarettes, equal to 24 puffs). Electrophysiological analysis of CFTR and BK channel activities using Ussing chambers (UC)^25^ with assessment of Cl^−^ and K^+^ fluxes across the apical membrane^10, 12, 25^, ASL volume as well as protein phosphorylation assays were performed 1, 2, 4 and 6 h post smoke exposure (Fig. 1a).

Cigarette smoke reduced apical CFTR and BK channel activities (Fig. 1b,c): Cigarette smoke decreased the CFTR and BK channel functions as early as 1 h after exposure compared to air control and these changes endured for the duration of the measurements. Results of cells from smokers and non-smokers are shown separately, indicating that these cells did not respond differently within the measured time frame. Even air exposure decreased CFTR activity measurably, which might be explained by evaporative loss^37^ (see discussion). A quantifiable loss in ASL volume was observed 2 h after smoke exposure and this loss persisted throughout the 6 h assay period when compared to control cells exposed to air only, again not different between cells from smokers and non-smokers (Fig. 1d). Transepithelial resistance (TER) did not change significantly in smoke- compared to air-exposed cells, assuring that observed Isc changes were not due to modulation of paracellular permeability (Fig. 1e).

### Cigarette smoke activates Smad3 and p38 signalling

**Figure 2:**
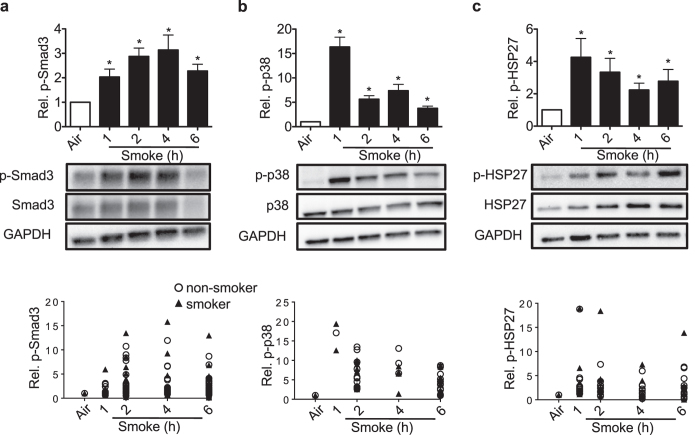
Smoke induces Smad3 as well as p38 and HSP27 phosphorylation. **(a)** Western blot quantification of Smad3 phosphorylation showed an >2-fold increase 1 h after smoke exposure, an about 3-fold at 2 h and 4 h as well as 2-fold increase at 6 h. (n ≥ 20 from 12 lungs). Below each quantification, a representative western blot is shown and below the blot, single data points of cells from smokers and non-smokers. **(b)** p38 phosphorylation was increased 16.3-fold upon smoke exposure within 1 h, followed by a decrease to ~3.8 fold at 6 h (n ≥ 10 from 10 lungs). Below each quantification, a representative western blot is shown and below the blot, single data points of cells from smokers and non-smokers. (**c**) HSP27 phosphorylation (n = 4 from 4 lungs) was increased within 1 h to ~4.5 fold and to ~2.5 fold at 6 h after cigarette smoke exposure. Below each quantification, a representative western blot is shown and below the blot, single data points of cells from smokers and non-smokers. All: Ratios were quantified for the appropriate phosphorylated protein as a fraction of total protein, corrected for GAPDH and normalized to air control. *Indicates p < 0.05 compared to control (6 h air exposure).

Exposure of HBECs to cigarette smoke resulted in activation of the Smad3 and p38 pathways. Cigarette smoke induced a persistent increase in Smad3 (Fig. 2a) and in p38 phosphorylation, even though p38 phosphorylation peaked to its highest level at 1 h (Fig. 2b). p38 activation was associated with phosphorylation of one of its target proteins, namely the small heat shock protein 27 (HSP-27; Fig. 2c). Smad3 and HSP27 phosphorylation did not significantly increase in HBECs exposed to air only (Supplementary Fig. S1). As was true for the MCC parameters CFTR, BK and ASL volume, protein phosphorylation followed the same pattern for cells from smokers and non-smokers (Fig. 2). Therefore, all experiments were all done with a mix of cells from smokers and non-smokers.

### Inhibition of Smad3 and p38 signalling ameliorates early cigarette smoke-induced CFTR and BK dysfunction

**Figure 3:**
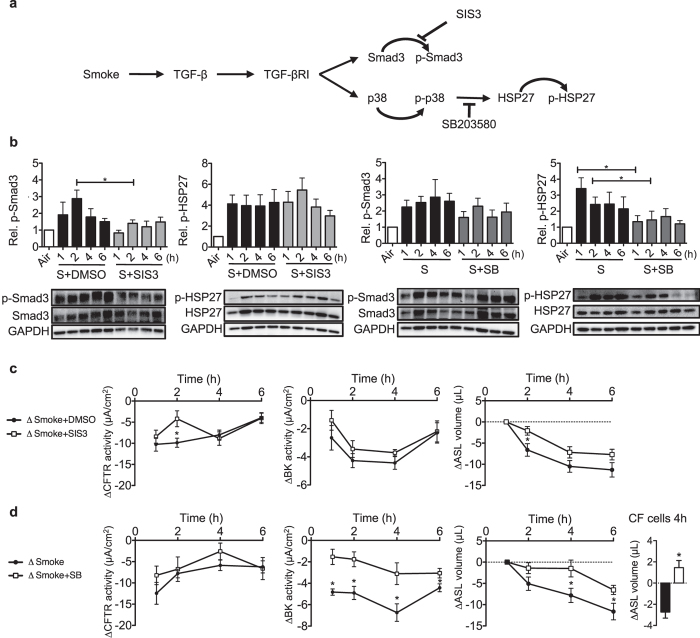
Changes in phosphorylation patterns and channel functions after smoke exposure in the presence of Smad3 and p38 signalling inhibitors. (**a**) Schematic diagram of the TGF-β pathway activated by smoke exposure with subsequent phosphorylation of Smad3 and p38/HSP-27. Appropriate inhibitors of different pathways are shown. (**b**) Smad3 phosphorylation was inhibited by SIS3 (3 µM) 2 h after smoke exposure, while SIS3 had no effect on HSP-27 phosphorylation. SB203580 (10 µM) had not effect on Smad3 phosphorylation but reduced HSP-27 phosphorylation 1 and 2 h after cigarette smoke exposure. Below each quantification, a representative western blot is shown. Abbreviations are: S (Smoke) and SB (SB203580). (**c**) SIS3 ameliorated decreases in CFTR conductance and ASL volume at 2 h after smoke exposure but had no effect on BK activity at any time point. (**d**) SB203580 did not rescue CFTR function at any time point, but BK activity was less decreased from 1 to 6 h after smoke exposure. ASL volume also did not decrease at 4 and 6 h. Finally, the importance of BK channels for ASL rescue was shown in CF cells 4 h after smoke exposure (far right). ΔCFTR and ΔBK activity represent the difference of smoke exposed and air exposed cells; ΔSmoke = Smoke-exposed cells - average of control cells exposed to air; ΔSmoke + DMSO/SB/SIS3 = Smoke + DMSO/SB/SIS3 - average of control cells exposed to air + DMSO/SB/SIS3. *Indicates p < 0.05 compared to control (air exposure). All n ≥ 4 from at least 3 lungs, except for the CF cells (duplicates measured in duplicates from one lung).

To identify whether Smad3 and/or p38 MAPK signalling contributed to CFTR or BK dysfunction and ASL volume loss, specific inhibitors for each pathway were used (Fig. 3a). HBECs were pre-treated for 1 h before cigarette smoke exposure with the Smad3 inhibitor SIS3 (3 μM) or the p38 inhibitor SB203580 (10 μM). SB203580 inhibits p38 MAPK catalytic activity by docking to the ATP-binding pocket (thus reducing its target HSP27 phosphorylation), but does not block phosphorylation of p38 MAPK itself by upstream kinases^38, 39^.

SIS3 significantly inhibited cigarette smoke-induced Smad3 phosphorylation 2 h post exposure but, as expected, SIS3 had no effect on HSP-27 phosphorylation (Fig. 3b). Conversely, the p38 inhibitor SB203580 had no effect on Smad3 phosphorylation but significantly decreased smoke-induced HSP-27 phosphorylation 1 and 2 h after cigarette smoke exposure (Fig. 3b).

In Ussing chamber experiments, SIS3 transiently improved CFTR activity 2 h post smoking (Fig. 3c left). SIS3 had no effect on smoke-induced BK channel dysfunction (Fig. 3c middle). Consistent with transient CFTR improvement at 2 h, SIS3 prevented ASL volume loss transiently at 2 h post smoke exposure (Fig. 3c right).

While SB203580 did not improve smoke-induced CFTR dysfunction (Fig. 3d left), it significantly ameliorated decreased BK activity upon smoke exposure, resulting in a concomitant modest increase in ASL volume at 4 and 6 h in cells from non-smokers and smokers (Fig. 3d). The importance of BK recovery for ASL volume homeostasis was also shown in cells from a CF patient; there, SB203580 recovered ASL volume 4 h after smoke exposure even in the absence of functional CFTR activity (Fig. 3d, right).

### LY2157299 decreases cigarette smoke-induced Smad3 and p38 phosphorylation and thereby improves CFTR and BK functions as well as ASL volume

**Figure 4:**
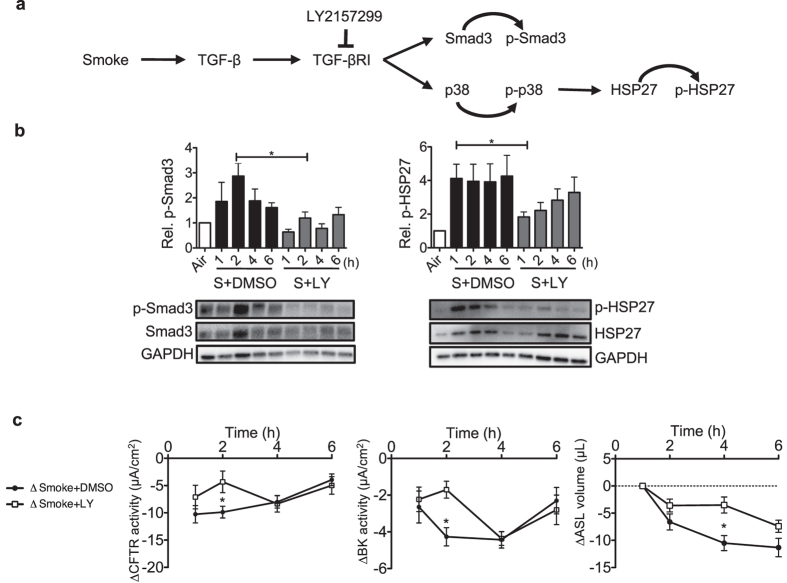
LY2157299 modulates TGF-β signalling after smoke exposure by decreasing Smad3 and p38 activation, thereby preserving CFTR and BK activities as well as ASL volume. (**a**) Schematic diagram showing LY2157299 (10 µM) effects on the TGF-β pathway (p38/HSP-27 and Smad3), activated by smoke exposure. (**b**) LY2157299 prevented smoke-induced Smad3 and HSP-27 phosphorylation at 2 h when compared to cells exposed to cigarette smoke alone. Below each quantification, a representative western blot is shown. Abbreviations are: S (Smoke) and LY (LY2157299). (**c**) LY2157299 improved CFTR and BK conductance at 2 h as well as ASL volume loss at 4 h after smoke exposure. ΔCFTR and ΔBK activity represent the difference of smoke exposed and air exposed cells, ΔSmoke + DMSO = Smoke + DMSO-exposed cells - average of control cells exposed to air + DMSO; ΔSmoke + LY = Smoke + LY2157299 - average of control cells exposed to air + LY. *Indicates p < 0.05 compared to control (6 h air exposure). All n ≥ 4 from at least 3 lungs.

Pre-treatment of HBECs with the LY2157299 (10 μM; Galunisertib), a selective TGF-β receptor I inhibitor (Fig. 4a), significantly inhibited Smad3 phosphorylation 2 h after smoke exposure compared to cells exposed to cigarette smoke alone (Fig. 4b left). LY2157299 also decreased smoke-induced HSP-27 phosphorylation 1 h post cigarette smoke exposure (Fig. 4b right). Consistent with SIS3 and SB203580, treatment with LY2157299 transiently increased CFTR and BK activities 2 h after smoke exposure in Ussing chamber experiments (Fig. 4c left and middle). LY2157299 also led to a moderate attenuation of smoke-induced ASL volume loss starting 4 h after exposure (Fig. 4c right). These results indicate that the TGF-β pathway is partially responsible for smoke-induced MCC dysfunction. ASL volume loss and both CFTR and BK dysfunction can be ameliorated, but not fully rescued with inhibitors of specific branches of TGF-β pathways.

### Pirfenidone counters cigarette smoke-induced p38 MAPK activation and limits BK dysfunction

**Figure 5:**
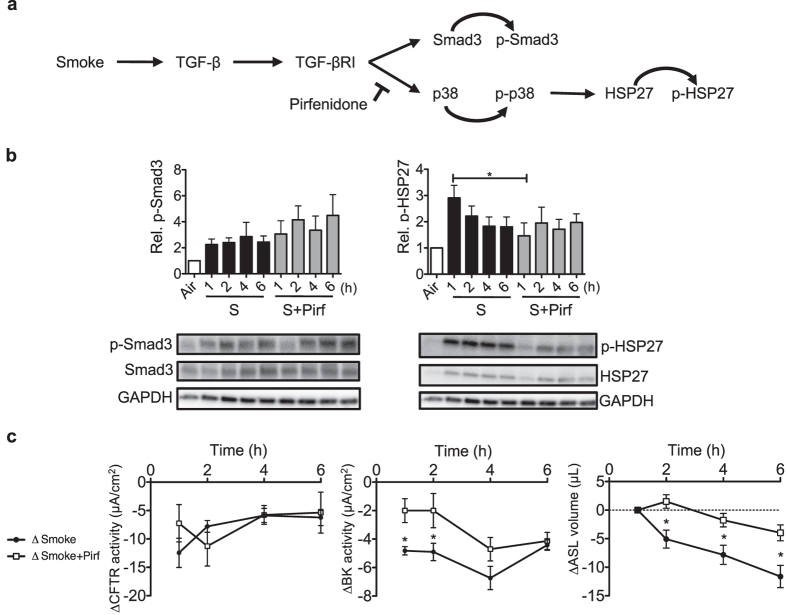
Pirfenidone transiently reduces smoke-induced p38 signalling, thereby ameliorating BK dysfunction and ASL volume loss. (**a**) Pirfenidone (1 mg/mL) inhibits smoke-mediated activation of p38 signalling. (**b**) Protein analysis using western blots showed no reduction of smoke-activated Smad3 phosphorylation by pirfenidone but a reduction of HSP27 phosphorylation 1 h after smoke exposure. Below each quantification, a representative western  blot is shown. Abbreviations are: S (Smoke) and Pirf (Pirfenidone). (**c**) Ussing chamber data showed that smoke-induced reduction in CFTR activity was not rescued by pirfenidone, but BK activity was temporarily improved at 1 and 2 h after cigarette smoke exposure. Pirfenidone also ameliorated smoke-induced ASL volume loss at 2, 4 and 6 h after exposure. ΔCFTR and ΔBK activity represent the difference of smoke-exposed compared to air exposed cells; ΔSmoke = Smoke-exposed cells - average of control cells exposed to air; ΔSmoke + Pirf = Smoke + Pirf - average of control cells exposed to air + Pirf. *Indicates p < 0.05 compared to control (6 h air exposure). All n ≥ 4 from at least 3 lungs.

Pirfenidone (5-methyl-1-phenyl-2-[1 H]-pyridone) is known to inhibit pulmonary fibrosis progression in animal models^40^ as well as in clinical trials^41–45^, at least in part by inhibiting TGF-β signalling (Fig. 5a). Pre-treating HBECs with 1 mg/ml of pirfenidone had no effect on Smad3 (Fig. 5b left), but decreased HSP-27 phosphorylation 1 h after cigarette smoke exposure (Fig. 5b right). While pirfenidone did not restore CFTR function, it improved BK channel activity 1 and 2 h after cigarette smoke exposure (Fig. 5c) and limited ASL volume loss 2–6 h after smoke exposure (Fig. 5c).

## Discussion

The data presented her show that during realistic cigarette smoke exposure of primary human bronchial epithelial cells (HBECs), inhibition of Smad3 phosphorylation reduced initial loss of CFTR activity, while inhibition of p38 MAPK reduced loss of BK activity. When either CFTR or BK function was at least temporarily maintained, the cells were able to maintain ASL volume for a limited period of time, indicating that both channels contribute to and are important for airway hydration.

Previous work from multiple laboratories, including our own, showed that smoke exposure of airway epithelial cells leads to CFTR dysfunction. However, this is the first report to show that cigarette smoke also decreases BK activity. In addition, this is the first report to show that partial rescue of BK activity after smoke exposure can temporarily ameliorate ASL volume depletion despite continued CFTR dysfunction in normal HBECs. While we have shown that BK rescue in cystic fibrosis (CF) cells can ameliorate TGF-β1-induced ASL volume loss, indicating the importance of BK channels, this is the first report to show that this is also the case in HBECs and CF bronchial epithelial cells when exposed to cigarette smoke. We also show for the first time that the outcome of short-term responses to cigarette smoke does not differ between cells obtained from smokers or non-smokers. Finally, the manuscript is based solely on realistic smoke exposure of the apical surface of fully differentiated cells, eliminating confounders seen by using *cell lines* and basolateral *cigarette smoke extract* exposure.

Our data provide evidence that realistic exposure to cigarette smoke using the VC-10 robot inhibits both CFTR and BK functions at the earliest measurement time point, namely 1 h after exposure. Subsequently, ASL volume depletion occurred 2 h after exposure and lasted throughout the measurement period of 6 h. Cigarette smoke did not significantly influence transepithelial resistance within this time frame, suggesting that changes in ASL volume were due to changes in ion channel function and not a result of compromised paracellular integrity.

The expression of TGF-β1 mRNA is significantly higher in smokers and COPD patients when compared to non-smokers^28^ (and Supplementary Fig. S4) and cells from smokers and non-smokers could respond differently to acute cigarette smoke exposure. In fact, baseline ASL volume was significantly decreased in HBECs from COPD patients compared to non-smokers (Supplementary Fig. S4a). Moreover, TGF-β1 expression is significantly higher and LRRC26 lower in cells from COPD patients at baseline (Supplementary Fig. S4b and c). On the other hand, changes upon acute smoke exposures were not different between cells from healthy smokers without COPD and non-smokers (Figs 1 and 2), indicating that pathways implicated in acute or short-term responses to cigarette smoke are similar in these cells. Thus, we used cells from smokers and non-smokers, excluding COPD donors.

Given the implication of TGF-β signalling in smoke exposure, we speculated that cigarette smoke-induced dysfunction of CFTR and BK channel activities could be mediated by distinct TGF-β pathways. In fact, cigarette smoke induced phosphorylation of both Smad3 and p38 MAPK occurred via TGF-β receptors since both could be inhibited with a TGF-β receptor blocker. Inhibition of Smad3 signalling improved CFTR function (Fig. 3C) while the p38 inhibitor SB203580 and pirfenidone ameliorated loss of BK activity (Figs 3D and 5C). ASL volume recovery was seen, at least temporarily, concomitantly with restoration of either one of these ion channel activities.

It is well described that cigarette smoke exposure induces CFTR alterations by gene transcription as well as mRNA or protein stability^26, 46^, leading to decreased CFTR activity and mucociliary dysfunction^47^. For instance, HBECs exposed to *cigarette smoke extract* showed reduced CFTR activity, ciliary beat frequency and ASL volume, all reversed by co-administration of the CFTR potentiator ivacaftor^48^. The here shown Smad3-dependent mechanism for early CFTR dysfunction was not always seen. Sun *et al*. demonstrated in cystic fibrosis epithelia that TGF-β-mediated activation of p38 MAPK caused CFTR downregulation^49^. In contrast, cigarette smoke-induced p38 activation had no effect on CFTR conductance, instead, the MEK/ERK/MAPK pathway regulated plasma membrane CFTR availability when measured upon 24 h *cigarette smoke extract* exposure^26^. While our results also showed ERK1/2 upregulation 1 h after cigarette smoke exposure (Supplementary Fig. S3), the TGF-β receptor I inhibitor LY2157299 did not modulate ERK1/2 signalling, but improved CFTR and BK activity.

Our results confirm the importance of proper BK channel function for ASL volume homeostasis^6, 9, 50^, even in the absence of functional CFTR. We previously described the negative direct effects of TGF-β1 treatment on BK activity and ASL volume in cells from cystic fibrosis patients, an effect mediated via downregulation of the BK γ subunit LRRC26^11^. Our results presented here indicate that LRRC26 mRNA levels significantly decreased 6 h post smoke exposure in HBECs from non-smokers (Supplementary Fig. S2) as one possible mechanism how smoke regulates BK activity, but clearly not the only one since BK dysfunction occurs earlier. On the other hand, we don’t have good assays to assess LRRC26 association with the α subunit of BK, which is critical for BK activity in non-excitatory cells. We speculate that non-association may precede LRRC26 mRNA/protein decreases.

Finally, the TGF-β inhibitor pirfenidone has been shown to block TGF-β-induced phosphorylation of Smad3 and p38 in primary human lung fibroblasts^51^. Our data show that pirfenidone blocked p38 phosphorylation and partially rescued BK channel activity while limiting ASL volume loss after smoke exposure. We showed previously that BK dysfunction was also rescued by pirfenidone upon TGF-β1 treatment in cells from cystic fibrosis patients^11^.

**Figure 6:**
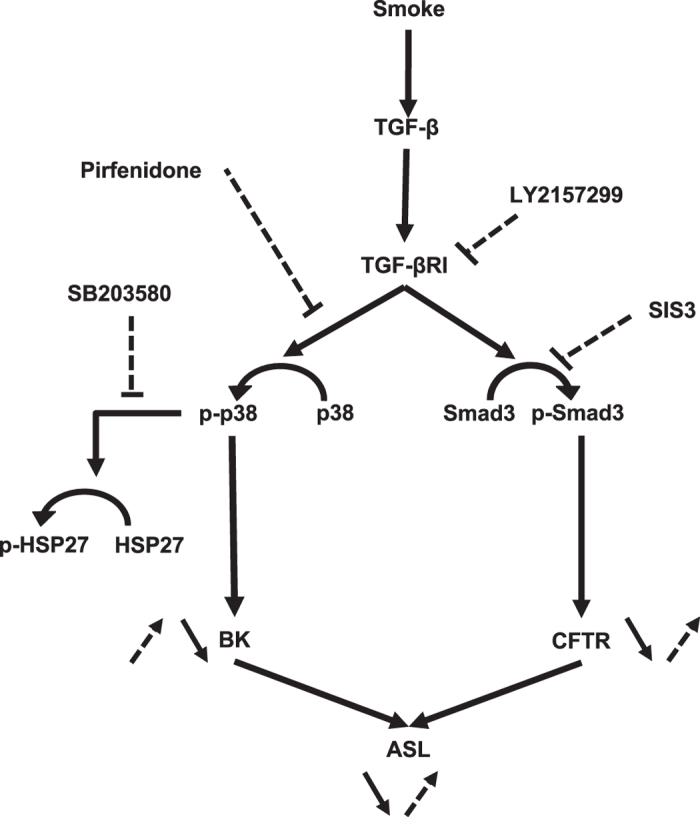
Schematic diagram of smoke effects on CFTR and BK activities as well as ASL volume in the absence or presence of different inhibitors. Smoke (black arrow), stimulates both Smad3 and p38 phosphorylation via TGF-β signalling, which in turn decreases CFTR and BK channel functions. Overall this causes ASL volume loss, resulting in mucociliary dysfunction. Inhibitors (dotted arrow) used for the presented experiments ameliorated CFTR and BK activities and thereby improved ASL volume loss.

In summary, we demonstrate that acute cigarette smoke exposure inhibits both CFTR and BK activities, leading to a decrease of ASL volume (Fig. 6). We identified different intracellular pathways involved in early cigarette smoke-induced channel dysfunction and showed that amelioration of ASL volume depletion was evident through transient restoration of either CFTR or BK function within 6 h post exposure, with both channels partially compensating for each other’s function. Since the anti-fibrotic drug pirfenidone restored ASL volume during the 6 h post cigarette exposure by improving BK function, it might represent a novel therapeutic strategy to ameliorate mucociliary clearance in patients with smoking-associated chronic bronchitis. Finally and not surprisingly, TGF-β signalling is not the only pathway activated to decrease CFTR and BK activities with ASL volume loss since none of the inhibitors was able to reverse these processes indefinitely.

## Methods

### Air-liquid interface (ALI) cultures

Normal human airways were obtained from organ donors whose lungs were rejected for transplant. Lungs were provided by the University of Miami Life Alliance Organ Recovery Agency and the LifeCenter Northwest, WA. Our Institutional Review Board (IRB) determined that consent for organ donation for research, obtained by the organ procurement agencies, covers research uses of this material. Thus, no additional approval was needed for the here carried out experimental protocols. cystic fibrosis (CF) cells were obtained from an appropriately consented CF patient at the time of transplant with IRB approved consent. No organs were procured from prisoners. However, all methods were carried out in accordance with relevant guidelines and regulations.

From these lungs, airway epithelial cells were isolated, de-differentiated through expansion on collagen I coated dishes and re-differentiated at the air-liquid interface (ALI) on collagen IV coated T-clear filters with 0.4 µm pores (Costar Corning, NY, USA) as previously described^33, 35, 36, 52, 53^. Briefly, de-differentiated cells were grown submerged for 4 or 5 days and then switched to the ALI when the cell layer was confluent. HBECs were used for experiments when they were fully differentiated, assessed by mucus production and ciliary beating, usually 3–4 weeks after establishing the ALI.

### Chemicals

Inhibitors were used from the following companies: SIS3 (Cayman #15945, Ann Arbor, MI, USA), SB203580 (Trocis #1402, Bristol, United Kingdom), LY2157299 (Selleckchem #S2230, Houston, TX, USA) and pirfenidone (Selleckchem #S2907, Houston, TX, USA).

### Smoke exposure of ALI cultures

Cigarette smoke exposure of cells grown at the ALI was accomplished with the Vitrocell VC-10 smoking robot (Vitrocell, Waldkirch, Germany), capable of whole smoke exposure of apical surfaces. HBECs were exposed to smoke from 4 cigarettes (24 puffs; 35 mL puff volume delivered every 60 seconds, ISO standard 3308) using Kentucky reference 3R4F cigarettes. Simultaneously, control cultures were exposed to ambient air only^8, 22^.

### Western Blotting

ALI cultured HBECs were lysed in RIPA buffer in the presence of protease inhibitors and cleared from debris by centrifugation. Proteins, 30 µg per lane, were separated using 10% SDS PAGE gels (Bio-Rad, Hercules, CA, USA) and electro-blotted onto PVDF membranes using the iBlot^®^2 (Invitrogen by ThermoFisher Scientific Inc. #IB24001, Waltham, MA, USA). Membranes were blocked with 5% BSA in PBS, 0.01% Tween 20, and incubated with the primary antibody overnight at 4 °C. After washing with PBS, 0.01% Tween 20, membranes were incubated with a secondary horseradish peroxidase-labelled antibody (KPL Inc., Gaithersburg, MD, USA). Signal was detected and quantified by chemiluminescence (ThermoFisher Scientific Inc., Waltham, MA, USA) using a ChemiDoc XRS system (Bio-Rad, Hercules, CA, USA). The membranes were stripped with Restore Western blot stripping buffer (ThermoFisher Scientific Inc., Waltham, MA, USA) and re-probed. Band intensities were quantified using Image Lab software (Bio-Rad, Hercules, CA, USA).

Primary antibodies: anti Smad3 (Cell Signaling #9523, Denvers, MA, USA), anti p-Smad3 (phospho S423+S425; Abcam #ab52903, Cambridge, United Kingdom), anti p-p38 MAP Kinase (Cell Signaling #9211), anti p38 MAPK (Cell Signaling #9212), anti p-HSP27 (Cell Signaling #2401), anti HSP27 (Cell Signaling #2402), anti ERK1/2 (Cell Signaling #4695 S) and anti p-ERK1/2 (Cell Signaling #9101 S) and anti-GAPDH (Santa Cruz #sc47724, Santa Cruz, CA, USA).

### Airway surface liquid (ASL) volume

ASL volumes from HBECs were quantified by meniscus scanning^54^. Scanned menisci data were analysed using the software generously provided by Dr. Myerburg (University of Pittsburgh).

### Electrophysiology

Fully differentiated HBECs on Snapwell filters were mounted in Ussing chambers (Easymount chamber; Physiologic Instruments) connected to a VCC MC6 voltage clamp unit (Physiologic Instruments, San Diego, CA, USA). Solutions were maintained at 37 °C by heated water jackets and bubbled with air. For BK activity, basolateral membranes were permeabilized for 30 min with 20 µM amphotericin B, 10 µM nigericin and 10 µM valinomycin because whole cell short circuit current recordings cannot distinguish K^+^ efflux (it measures net current, a combination of K^+^ and Cl^−^ efflux)^10^. Furthermore, cells were exposed to a basolateral (140 mM) to apical (5 mM) K^+^ gradient, in the presence of apically applied 10 µM amiloride (Sigma-Aldrich #A7410, St. Louis, MO, USA) and 10 µM ATP (Sigma-aldrich #A1852). CFTR activity was measured with apical 5 mM Cl^−^ in the presence of apically applied 10 µM amiloride, 10 µM forskolin (Sigma-Aldrich #F3917) followed by 10 µM CFTR_inh_172 (Sigma-Aldrich #C2992)^8^.

### Transepithelial Resistance (TER) measurements

In Ussing chambers, input resistance of each filter was intermittently measured by 1 mV bipolar pulses of 2-second duration.

### Statistics

ANOVA or paired and unpaired t-tests (as appropriate) were used to compare data using Prism software (GraphPad Software, La Jolla, CA, USA) with a p < 0.05 accepted as significant.

### Data availability

All data generated or analysed during this study are included in this published article (and its Supplementary Information files).

## Electronic supplementary material

Supplementary Data
