# Supplementary material for: Role of Smad3 and p38 Signalling in Cigarette Smoke-induced CFTR and BK dysfunction in Primary Human Bronchial Airway Epithelial Cells
Source: Sci Rep. 2017 Sep 5;7:10506. doi: 10.1038/s41598-017-11038-x (PMC5585359; doi:10.1038/s41598-017-11038-x)

Supplementary materials for

Role of Smad3 and p38 Signalling in Cigarette Smoke-induced CFTR and BK dysfunction in Primary Human Bronchial Airway Epithelial Cells

Juliette Sailland, Astrid Grosche, Nathalie Baumlin, John S. Dennis, Andreas Schmid, Stefanie Krick, and Matthias Salathe*

Supplemental methods

Quantitative PCR: Total RNA was extracted using an RNeasy Plus Mini Kit (Qiagen, Valencia, CA) and reverse-transcribed into cDNA using the iScript cDNA synthesis kit (Bio-Rad, CA, USA) according to the manufacturer's instructions. Quantitative PCR (qPCR) was performed using a TaqMan Universal PCR Master Mix (Applied Biosystems) with the TaqMan Gene Expression Assays (Life Technologies, Grand Island, NY, USA). The following assays were used: LRRC26 (HS02385555_g1), TGF-β1 (HS00998133_m1) and GAPDH (4352934E). The difference in the threshold cycle between the targeted gene and the housekeeping gene GAPDH (X1000) was used as an estimation of the relative level of expression.

Supplemental figures legends

Supplemental Figure S1: Effect of inhibitors on Smad3 and p38 signaling in HBECs. Quantification of Smad3 and HSP27 phosphorylation showed that neither SB203580 (**a**), pirfenidone **(a),** LY2157299 (**b**) nor SIS3 **(b)** significantly changed baseline phosphorylation after air exposure (all $p>0.05$). Below each quantification, a representative Western blot is shown.

Abbreviations are: A (Air), SB (SB203580), Pirf (Pirfenidone) and LY (LY2157299). All $n \geq 4$ from at least 3 lungs.

Supplemental Figure S2: Effect of smoke on LRRC26 mRNA expression. Smoke significantly decreased LRRC26 mRNA expression at the 6h mark. * indicates $p < 0.05$ compared to control (6h air exposure). All $n \geq 4$ from at least 3 lungs.

Supplemental Figure S3: Effect of smoke on ERK1/2 and ERK1/2 phosphorylation in the absence or presence of LY2157299. (a) Smoke-induced ERK1/2 phosphorylation (ERK1/2-p/total ERK1/2) was detectable with an increase from 1 to 6h after smoke exposure. LY2157299 (10 μM) did not significantly decrease ERK1/2 phosphorylation ($p>0.05$). **(b)** Air+DMSO and air+LY2157299 did not differ with respect to ERK1/2 phosphorylation. Abbreviations are: A (Air), S (Smoke) and LY (LY2157299).

Supplemental Figure S4: Baseline ASL volume, TGF-β1 and LRRC26 mRNA levels are decreased in HBECs from patients with COPD compared to non-smokers. (a) ASL volume is significantly decreased in cell cultures using cells from COPD patients compared to non-smokers. **(b)** Quantitative PCR shows that TGF-β1 mRNA levels are increased and **(c)** LRRC26 mRNA levels decreased in HBECs from patients with COPD compared to non-smokers. mRNAs are relative to GAPDH (X1000). * indicates $p < 0.05$ compared to control (non-smokers). All $n \geq 4$ from at least 3 lungs. For the data shown in the main paper therefore, no cells from lungs with COPD were used.

At the end, all original Western blots are shown.

Figure s1

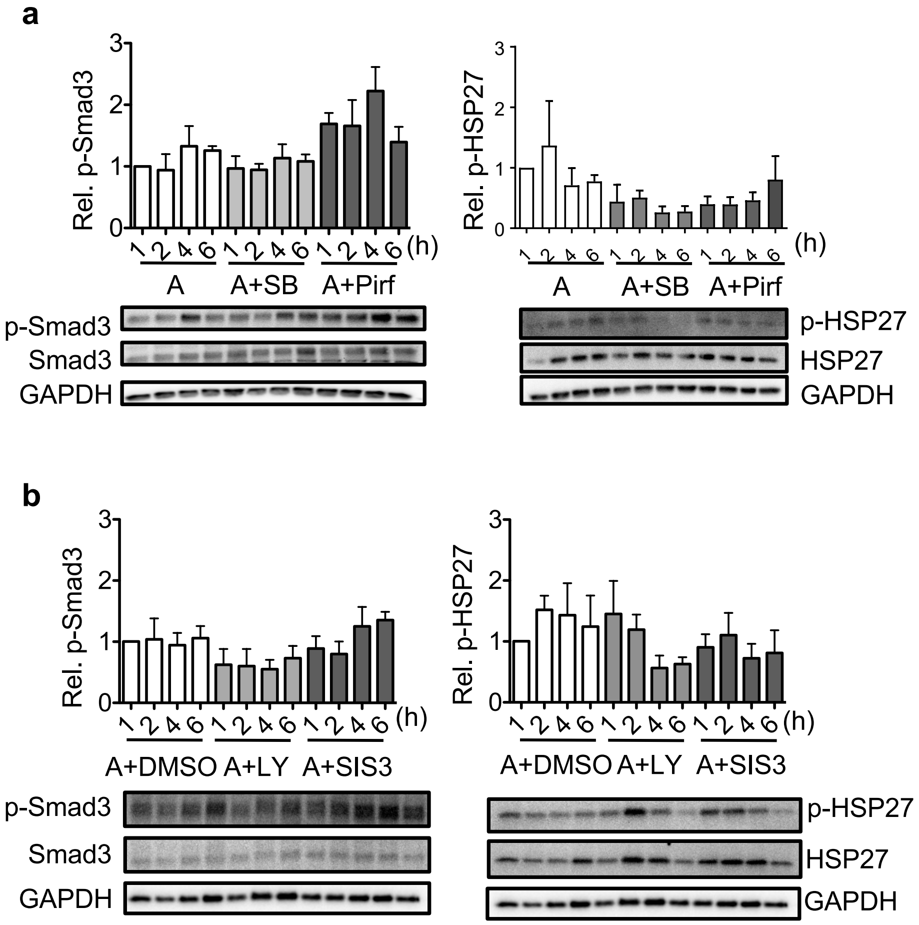
a
Rel. p-Smad3
3
2
1
0
1 2 4 6 (h)
A
A+SB
A+Pirf
p-Smad3
Smad3
GAPDH
Rel. p-HSP27
p-HSP27
HSP27
GAPDH
b
Rel. p-Smad3
A+DMSO
A+LY
A+SIS3
p-Smad3
Smad3
GAPDH
Rel. p-HSP27
p-HSP27
HSP27
GAPDH

Figure s2

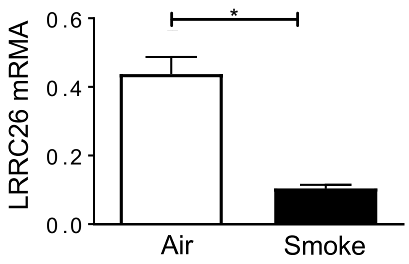

Figure s3

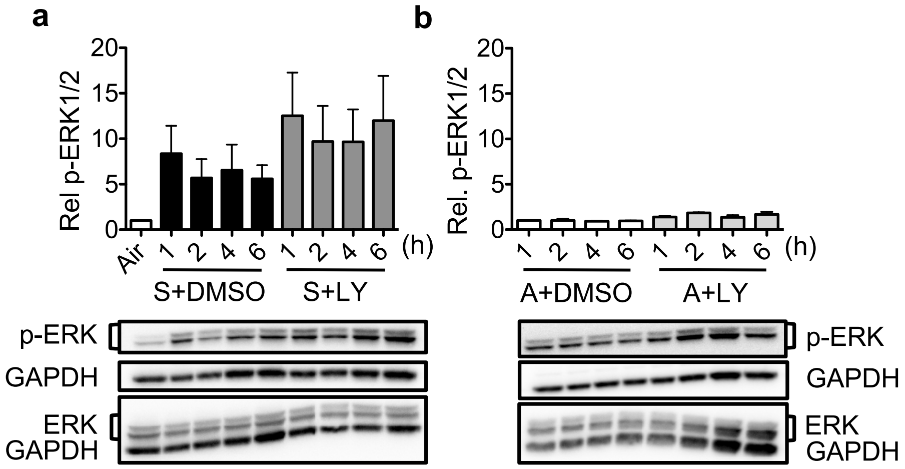

a
b
Rel p-ERK1/2
Rel. p-ERK1/2
0
5
10
15
20
Air
1
2
4
6
(h)
S+DMSO
S+LY
A+DMSO
A+LY
p-ERK
GAPDH
ERK
GAPDH

Figure s4

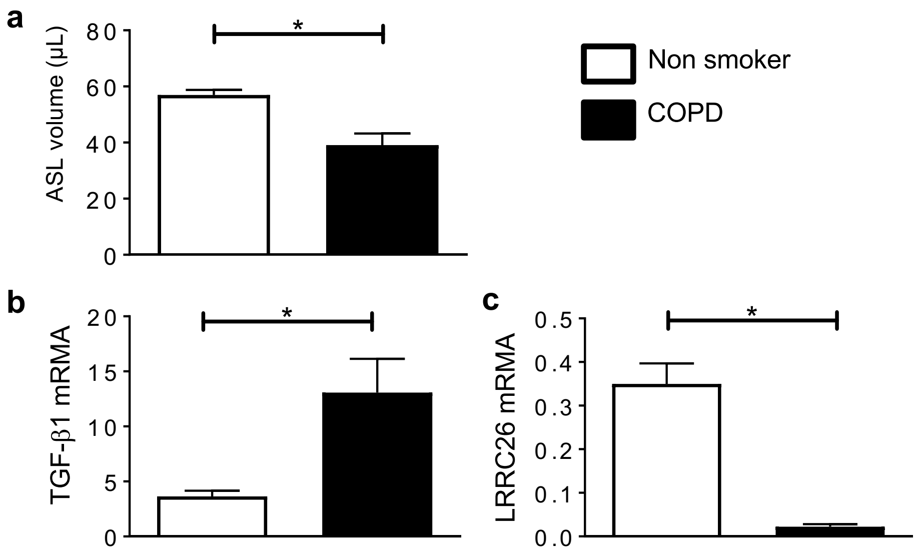
a
ASL volume (μL)
80
60
40
20
0
*
Non smoker
COPD
b
TGF-β1 mRMA
20
15
10
5
0
*
c
LRRC26 mRMA
0.5
0.4
0.3
0.2
0.1
0.0
*

Full images of Western bots for Figure 2

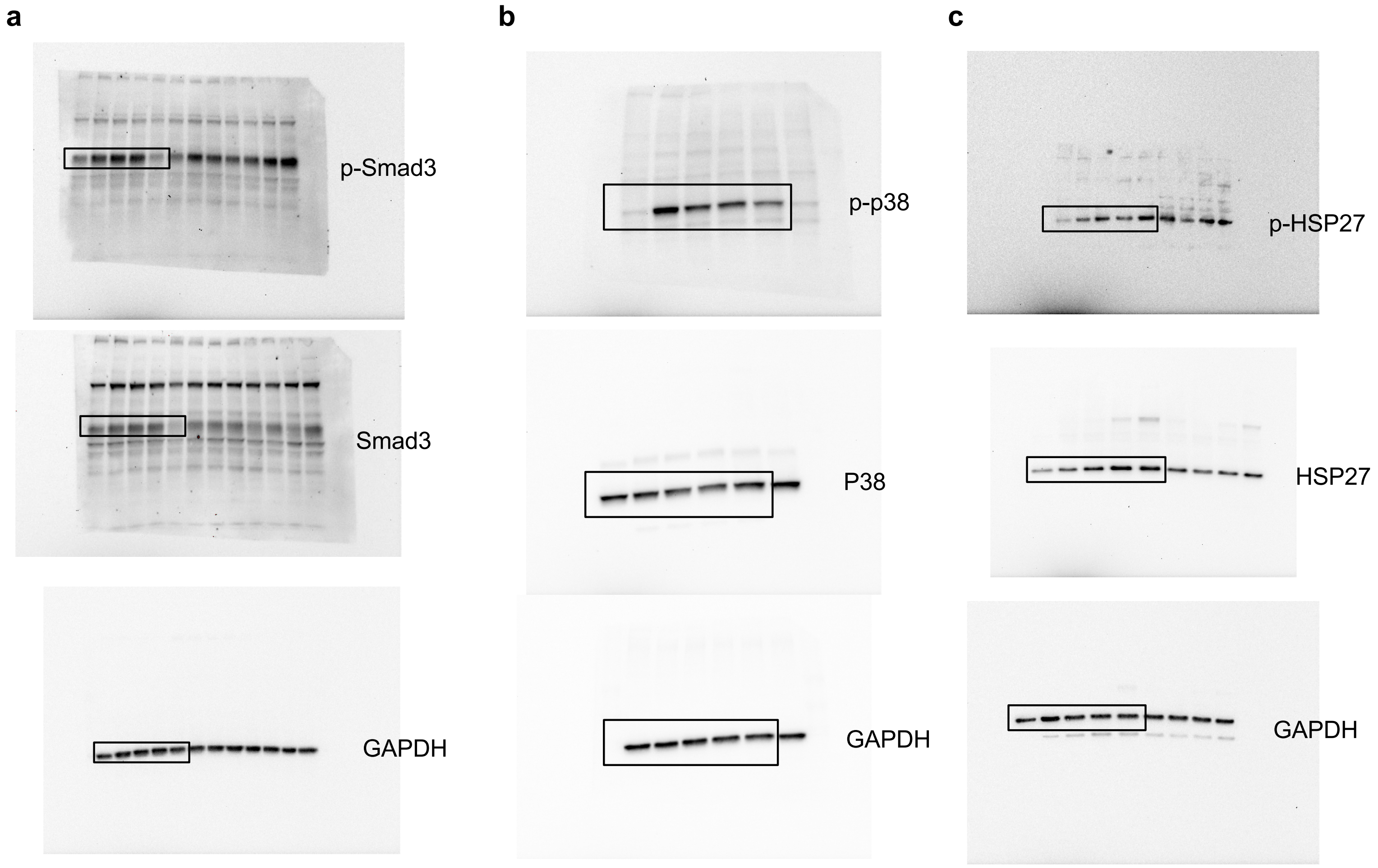

Full images of Western bots for Figure 3

b left two panels

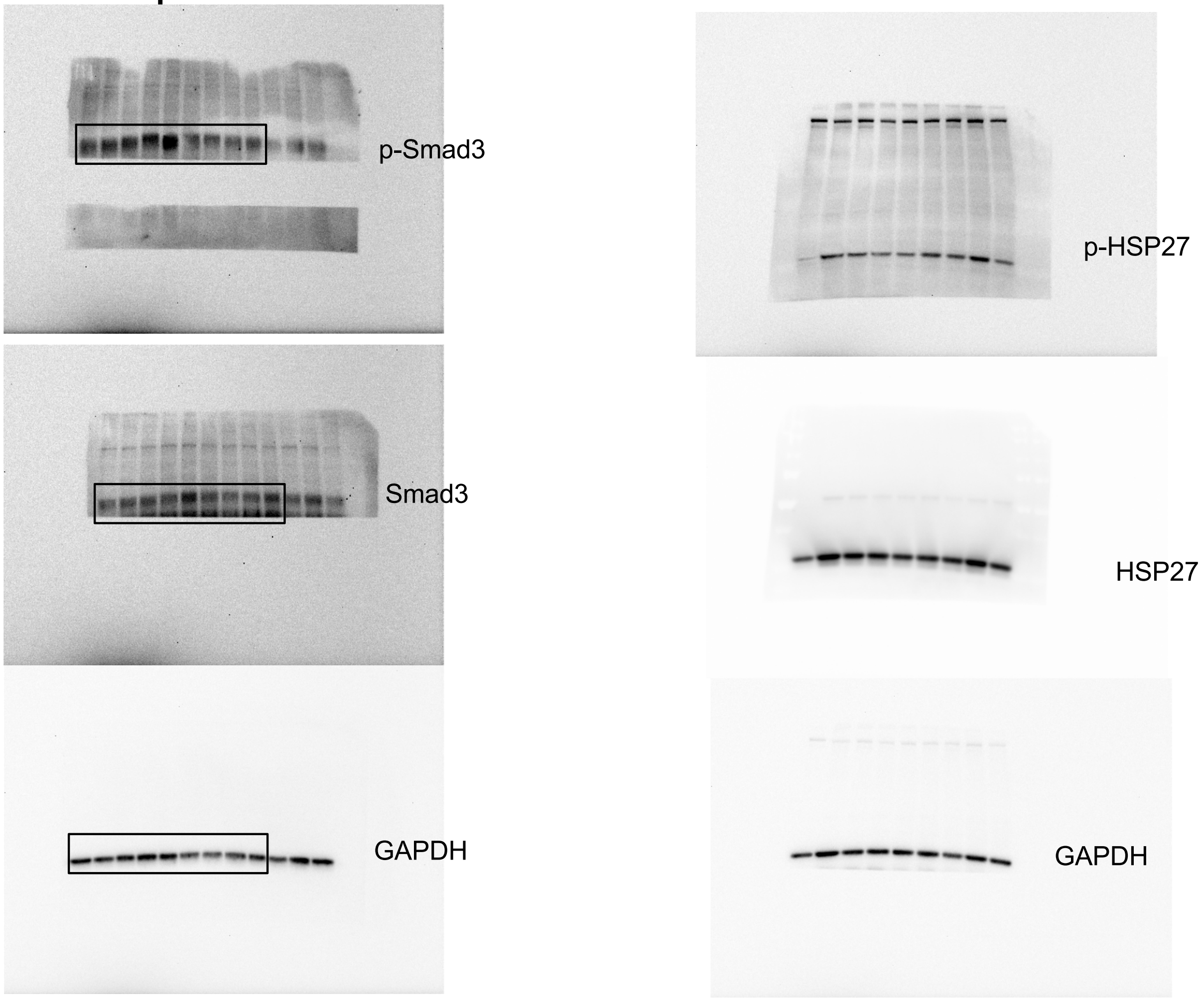

b right two panels

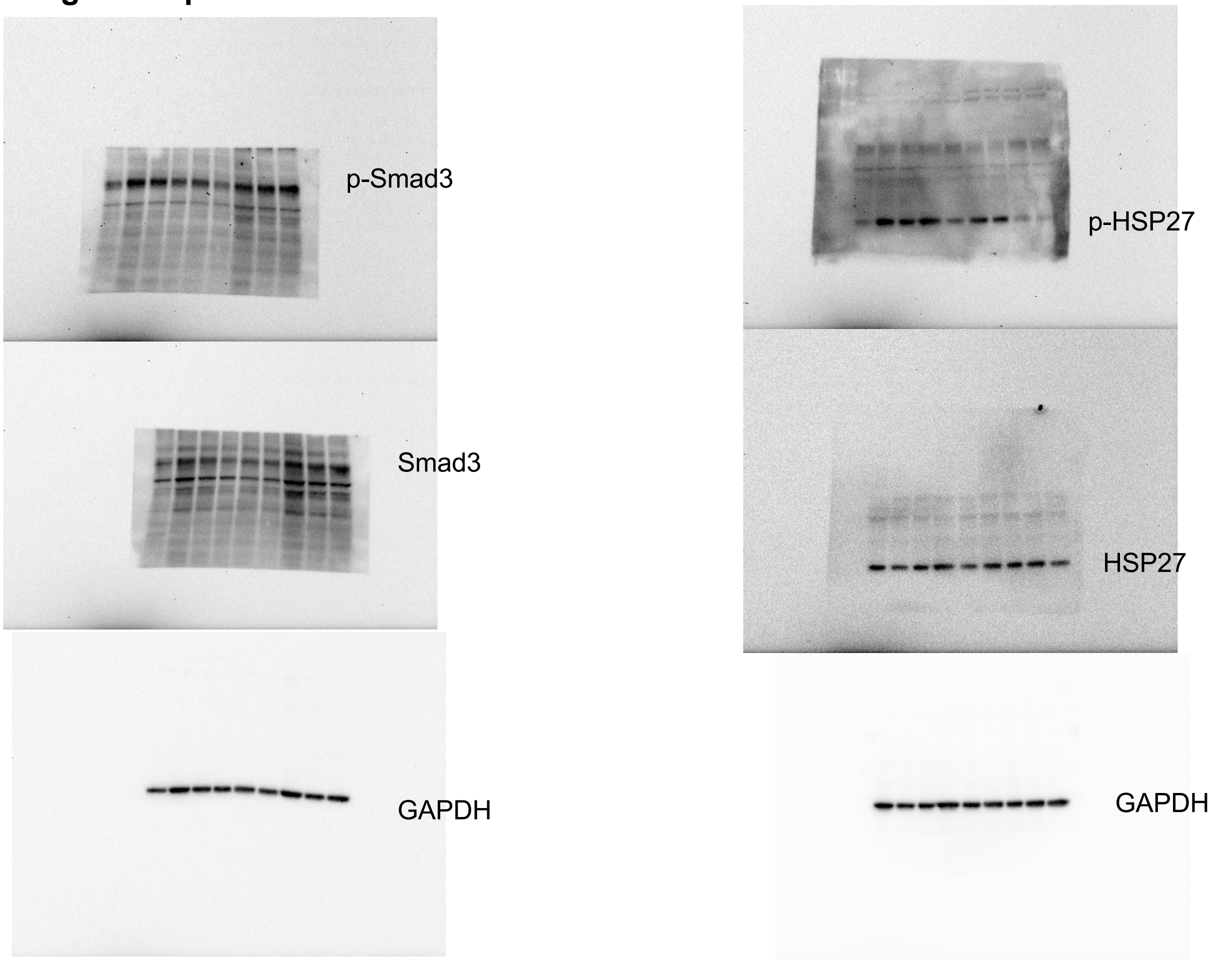

Full images of Western bots for Figure 4

a

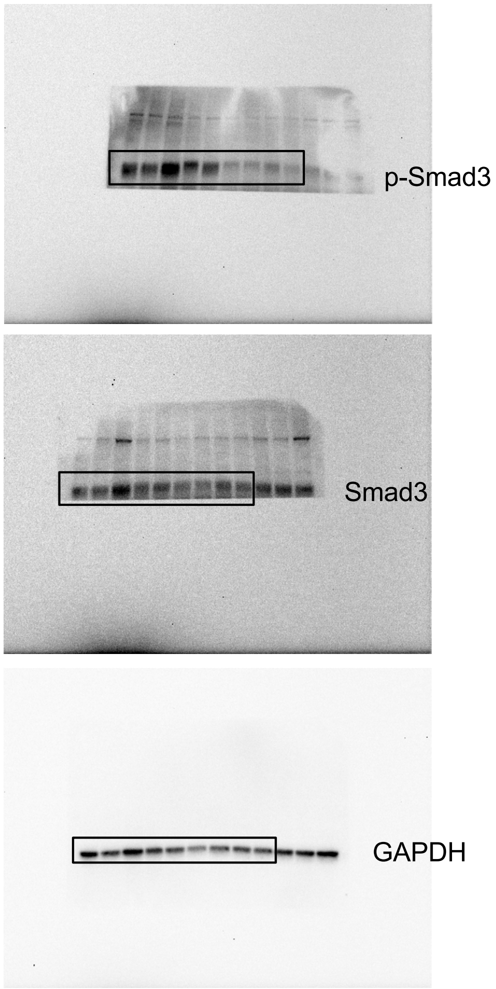

b

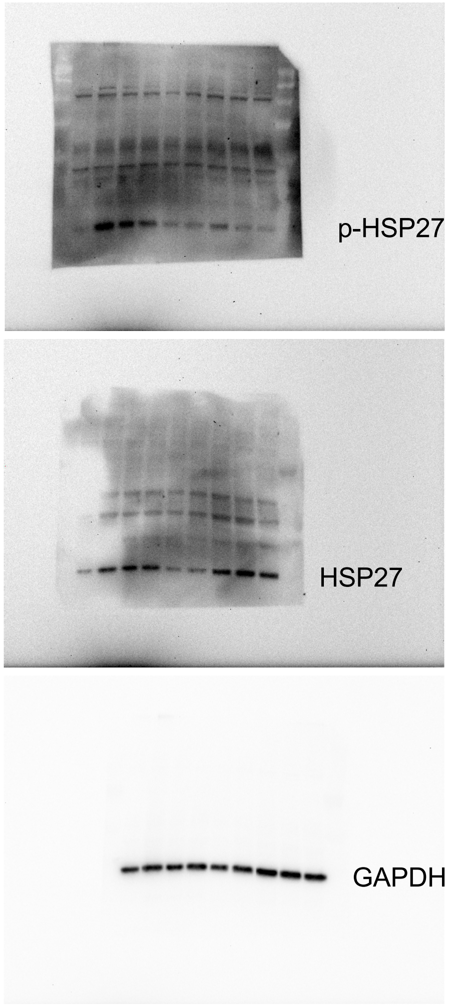

Full images of Western bots for Figure 5

a **b**

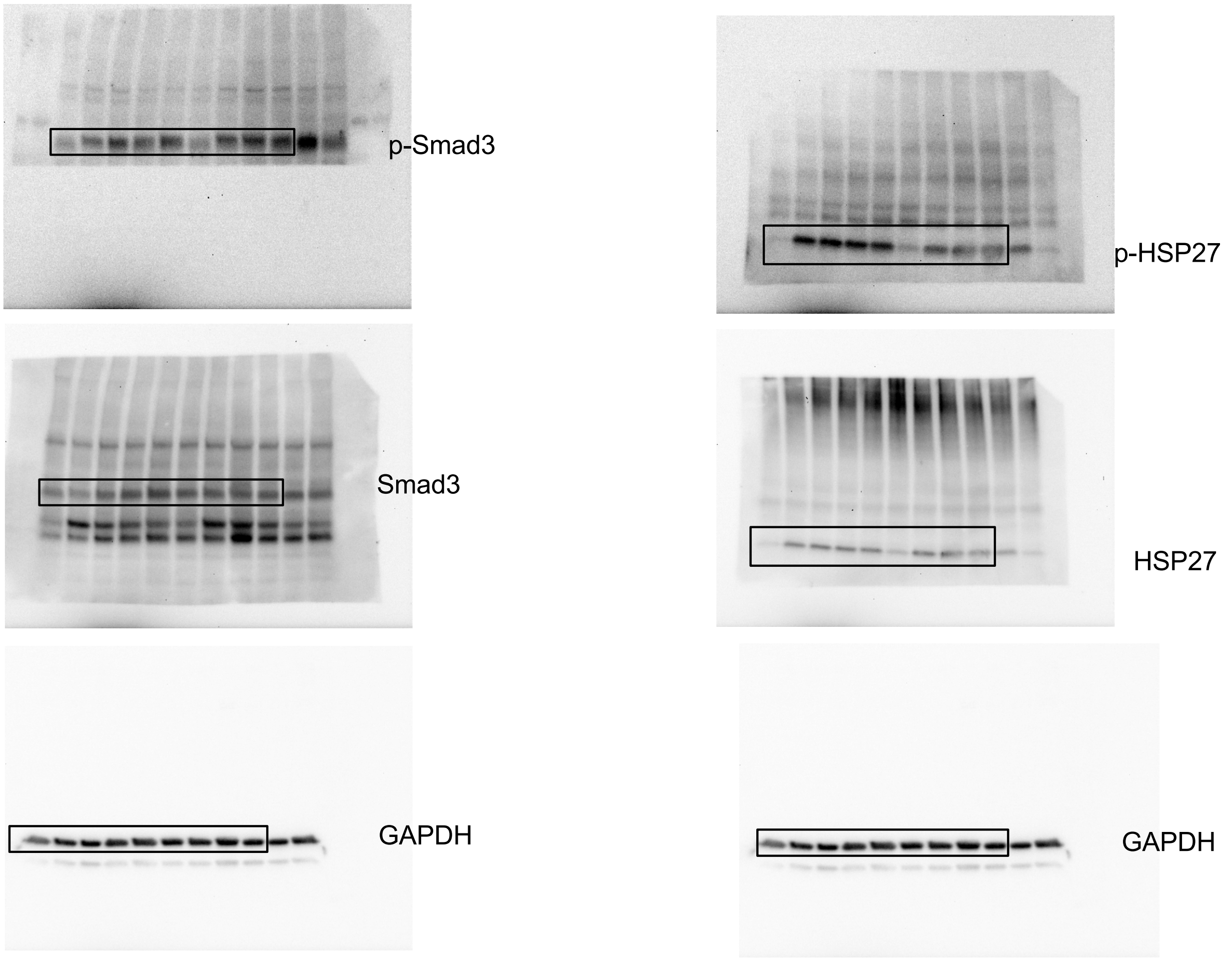

Full images of Western bots for Figure S1

a

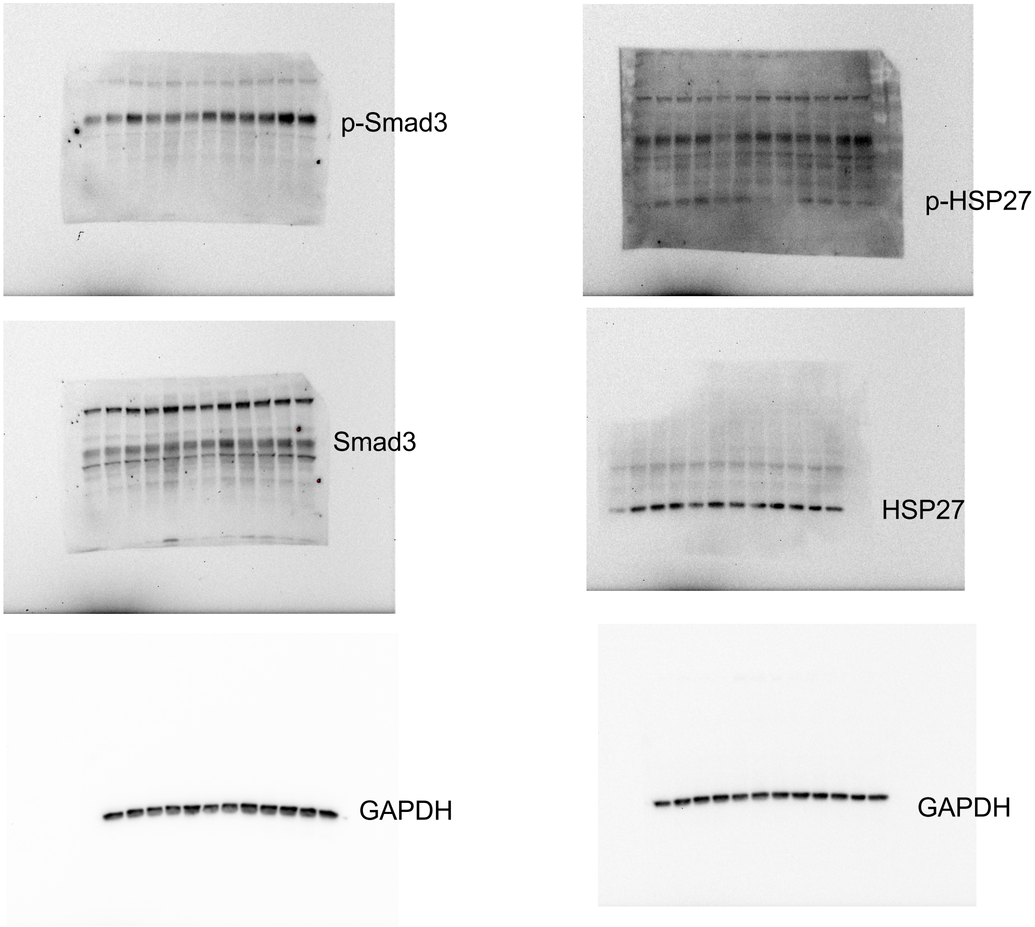

b

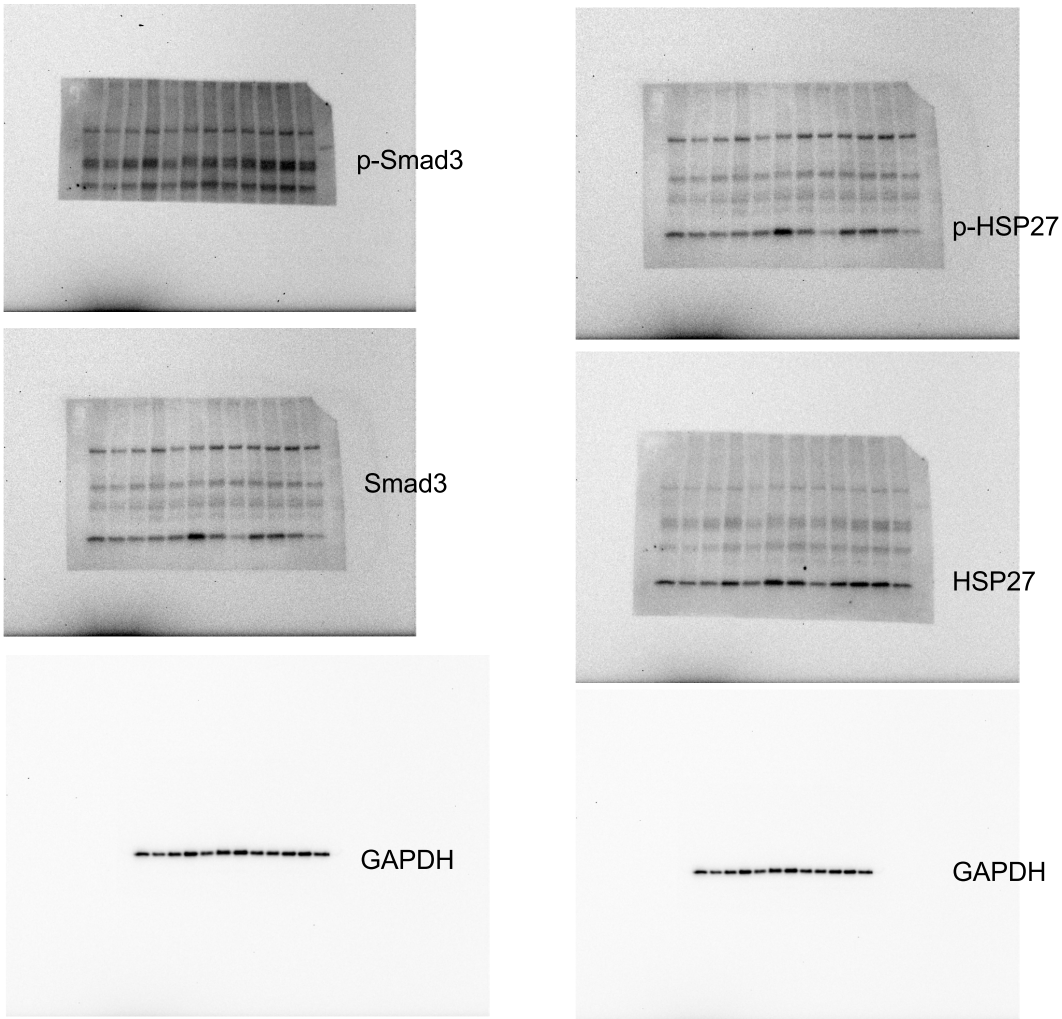

Full images of Western bots for Figure S3

a

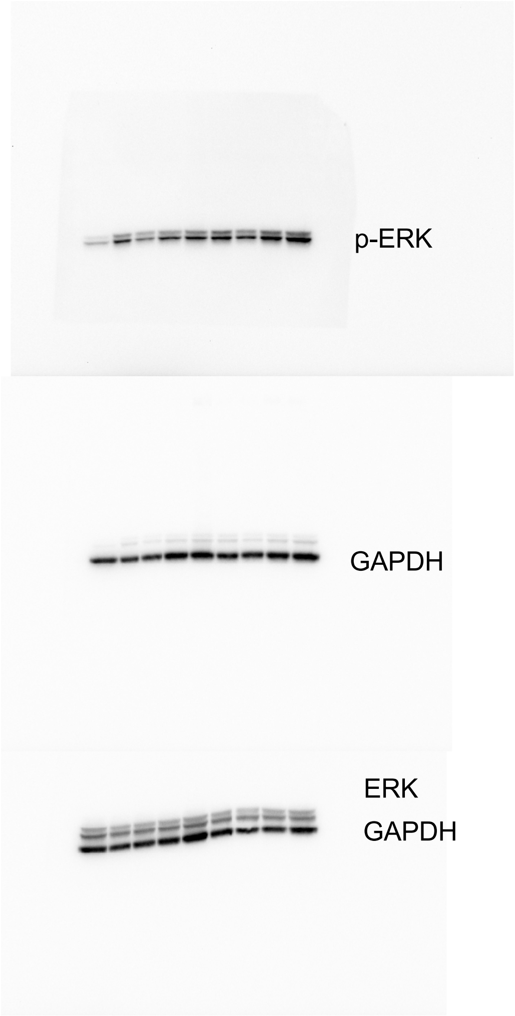

b

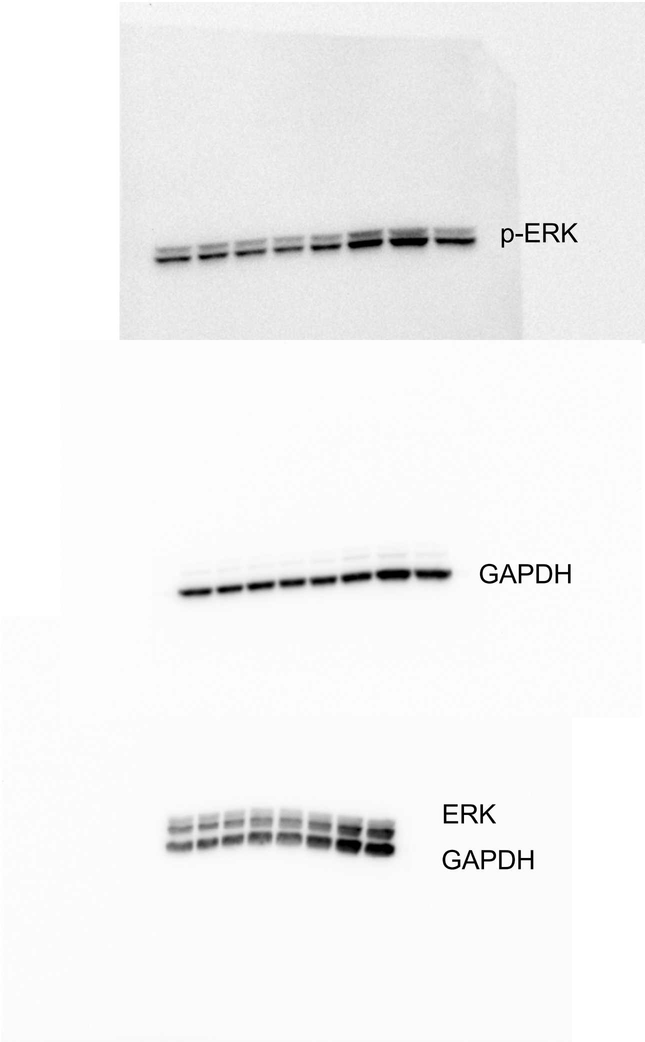

Supplement: Supplementary file 1 — Supplementary Data [file 41598_2017_11038_MOESM1_ESM.pdf]
